# An Effective Two-Step Procedure Allowing the Retrieval of the Non-Redundant Spherical Near-Field Samples from the 3-D Mispositioned Ones

**DOI:** 10.3390/s25185626

**Published:** 2025-09-09

**Authors:** Francesco D’Agostino, Flaminio Ferrara, Claudio Gennarelli, Rocco Guerriero, Massimo Migliozzi, Luigi Pascarella

**Affiliations:** Dipartimento di Ingegneria Industriale, Università di Salerno, Via Giovanni Paolo II, I-84084 Fisciano, Italy; fdagostino@unisa.it (F.D.); flferrara@unisa.it (F.F.); rguerriero@unisa.it (R.G.); mmigliozzi@unisa.it (M.M.); lpascarella@unisa.it (L.P.)

**Keywords:** antenna measurements, correction of probe mispositioning errors, near- to far-field transformations, non-redundant sampling representation, spherical near-field measurements

## Abstract

In this article, a novel procedure is developed to properly handle the 3-D mispositioning of the scanning probe in the near-field to far-field (NFtFF) transformations with spherical scanning for quasi-planar antennas under test, which make use of a non-redundant (NR) number of samples. It proceeds through two stages. In the former, a phase correction technique, named spherical wave correction, is applied to compensate for the phase shifts of the collected NF samples, which do not belong to the measurement sphere, due to mechanical defects of the arc, or inaccuracy of the robotic arm employed in the considered NF facility driving the probe. Once the phase shifts have been compensated, the recovered NF samples belong to the set spherical surface, but their positions differ from those prescribed by the adopted NR representation, because of an imprecise control and/or inaccuracy of the positioning system. Thus, the resulting sampling arrangement is affected by 2-D mispositioning errors. Accordingly, an iterative procedure is used in the latter step to restore the NF samples at their exact locations from those determined at the first step. Once the correct sampling arrangement has been retrieved from the 3-D mispositioned one, an optimal sampling interpolation formula is employed to obtain the massive input NF data necessary for the classical spherical NFtFF transformation technique. Numerical results, showing the precision of the NF and FF reconstructions, assessed the efficacy of the developed procedure.

## 1. Introduction

Antenna near-field (NF) measurements and the corresponding NF to far-field (NFtFF) transformation techniques [1,2,3,4,5,6,7,8,9,10,11] are becoming increasingly recognized practices for accurately assessing and quantifying antenna radiation performance. Indeed, they can be conducted within an anechoic chamber, a nearly reflection and interference-free environment allowing the suppression of multipath and the elimination of the effects due to weather. They are used for very large antennas for which the FF distance becomes too large to fit within it, or in an actual FF test range. NF measurements have the advantage of allowing high accuracy in the determination of the antenna FF pattern and permit a complete characterization of the antenna under test (AUT) performance, providing the gain, pattern, polarization, beam pointing, etc. They are usually collected through planar, cylindrical, or spherical setup at proper raster grid points lying on a plane, a cylinder, or a sphere surrounding the AUT and then post-processed to finally get its radiated FF pattern. The post-processing entails wave expansions of the electromagnetic (EM) field corresponding to the scanning surface.

Spherical near-field (SNF) measurements are significantly beneficial as compared to those gathered in planar and cylindrical scan geometries, since their post-processing, through NFtFF transformations [12,13,14,15,16,17,18,19,20,21,22,23,24,25,26,27,28,29,30,31,32,33,34,35,36], possesses the distinctive capability to obtain the whole radiation pattern of the antenna from a single set of NF measurements, thereby mitigating errors associated with the truncation of the scanning area. The standard spherical NFtFF [16] requires that the samples be gathered on a raster grid at the intersection points of regularly spaced parallels and meridians, whose numbers depend on the radius of the smallest sphere enclosing the antenna (minimum sphere rule). Then, these NF samples are transformed, through the spherical wave (SW) expansion, into the radiated AUT far field that accordingly is represented as a sum of spherical waves, each having specific modes. Anyhow, in accordance with the minimum sphere rule, the larger the AUT maximum dimension, the larger the amount of required samples and the longer the times for their collection. 

To relieve such a burden, novel approaches, which leverage the adaptive sampling [29], the compressive sensing [30,32,35,36], the non-redundant (NR) sampling representations of EM field [17,25,27,28] (whose theoretical foundations are detailed in [11,37]), have been developed in recent years. In particular, the latter appear very attractive, since they share the same framework of the standard NFtFF transformation [16], exhibit analogous mechanical complexity, thus avoiding any change in an existing SNF facility, and do not enhance the involved numerical complexity. According to the results in [11,37], they prescribe to model the AUT as efficiently as possible to achieve a truly NR sampling arrangement, characterized, unlike that of the standard SNF scan, by a reduced set of measurement points non-uniformly distributed over the scan sphere and, accordingly, capable to shorten the acquisition times. Thus, the geometry of the AUT represents the a priori information in the choice of the convex surface modeling it able to reduce the volumetric redundancy resulting from the minimum sphere rule. To this end, prolate spheroids [11,17,27] or rounded cylinders [11,25,28] may allow the best fitting of long AUTs; whilst oblate spheroids [11,17,27] or double bowls [11,25,28] may be used to suit quasi-planar AUTs. Then, the measured NR data are interpolated via a 2-D optimal sampling interpolation (OSI) formula for retrieving the numerous NF data at the points of the classical SNF raster grid [16], thus enabling the application of the standard NFtFF for the computation of the AUT radiated far field.

However, the accuracy of the FF reconstruction can be severely impaired by how precisely the acquisition points are reached. In fact, achieving the prescribed sampling points may be precluded by the poor accuracy of the positioners and/or by their inaccurate control, thus giving rise to 2-D positioning errors of the measuring probe. Moreover, when the collection of the spherical NF measurements is made by guiding the probe through an arc, defects due to mechanical manufacturing may be reflected in departures from the set scanning surface. The same may occur when the scanning is made by using a robotic arm. As a result, these two sources of positioning errors combine, resulting in 3-D positioning errors. Anyhow, the actual positions of the probe can be read by laser devices, thus allowing one to quantify the amount of such errors that, consequently, can be assumed known. Note that these errors can be heavier as the working frequency increases, as in the case of the fifth and sixth generations of cellular technologies. In fact, in such a case, even small mechanical flaws and inadequate resolutions of the involved positioners can reflect in more significant FF reconstruction errors. Accordingly, the availability of techniques capable of correcting these positioning errors, which prohibitively compromise the accuracy of the AUT characterization process, is nowadays perceived as a necessity. To this end, approaches relying on the matrix formulation have been proposed with reference to the classical plane—rectangular [38], cylindrical [39], and spherical [39,40] NFtFF transformations. Anyhow, these approaches are not suitable for the NFtFF transformation techniques relying on the NR theoretical background, which adopts sampling arrangements quite different from those for the standard NFtFF transformations.

Procedures for the compensation of 3-D probe positioning errors corrupting the NR NF measurements collected within a plane—rectangular and cylindrical NF facilities have been devised in [41,42,43]. In both cases, the underlying hypothesis is that the correction of errors related to the deviations of the measurement points from the set scanning surface and that due to the mispositioning with respect to prescribed NR sampling points on the surface can be treated as two independent problems. The devised two-step procedures take properly into account this hypothesis. Thus, the former step performs a suitable phase correction to compensate for the phase error caused by the deviations from the set scan surface. The so-called k correction [44] is employed in [41] to correct the deviations from the set measurement plane affecting the NF data collected through the NR plane-rectangular scan, whereas a cylindrical wave (CW) correction, resembling the k correction, has been devised to correct the radial errors affecting the NR cylindrical [42] and helicoidal [43] measurements. After the phase correction step, the NF samples belong to the set scan surface, although each of them result to be mispositioned with respect to the corresponding sampling point prescribed by the used NR representation, but close to it. The related errors are then accounted for in the latter step by resorting to efficient iterative techniques [41,42,43], which allow one to retrieve the NR data that would have been sampled at the points of the corresponding NR sampling grids in a positioning error free environment. Once the NR data have been retrieved at the correct positions of the NR grids, then they can be interpolated via the corresponding OSI formula to recover the NF data necessary for the standard NFtFF transformations.

The aim of this article is to extend this two-step procedure to the case of the NR NFtFF transformations with spherical scanning for quasi-planar antennas. In devising the phase correction involved in the first step of the procedure, it must be noted that both the k and CW corrections fail. In fact, the k correction is valid in the hypothesis that all the NF energy propagates in the main beam direction of the AUT, which is the same hypothesis underlying the plane wave expansion [41,44]. Whilst, the CW correction has been adopted to match the basic hypothesis of the standard cylindrical NFtFF transformation that most of the NF energy of a long AUT (usually characterized within a cylindrical facility) is propagating in the directions transverse to its longitudinal dimension [42,43]. As the SNF measurements are used to obtain, via an SW expansion, the FF pattern over the entire FF sphere, then a SW correction is proposed to compensate for the deviations from the nominal scan surface. The second step, wherein the NF samples lie on it, but are corrupted by 2-D positioning errors, proceeds with the adoptions of the iterative algorithm [45,46] to restore the NR data at their correct positions. It must be stressed that, to the best of the authors’ knowledge, the paper is the first to address the problem of NFtFF transformations from NR spherical NF data when the scanning process is affected by 3-D probe mispositioning errors. In fact, although other techniques have considered probe misplacement in redundant or oversampled measurement schemes, no previous contribution has tackled this issue in the framework of NR spherical scanning.

The paper is structured as follows. Section 2 reports the fundamental steps to develop a NR sampling representation of the voltage acquired via a non-directive probe over the scan sphere, based on the use of either an oblate spheroid or a double bowl to model quasi-planar AUTs. The OSI formula, utilised for the precise recovery of the probe voltage at any point on the measurement sphere from a NR number of its samples, is also shown. In Section 3, the two-step approach devised for correcting the 3-D probe mispositioning errors is thoroughly described. Section 4 presents numerous simulation results, which assess the performance and the reliability of the approach. Finally, Section 5 contains the concluding remarks.

## 2. Development of a NR OSI Representation on a Sphere

The main results concerning the development of an efficient representation of the probe voltage on a spherical scanning surface from a NR set of its uniformly distributed NF samples, optimised for quasi-planar AUTs, as well as the associated OSI expansion, are here outlined for the reader’s convenience.

**Figure 1:**
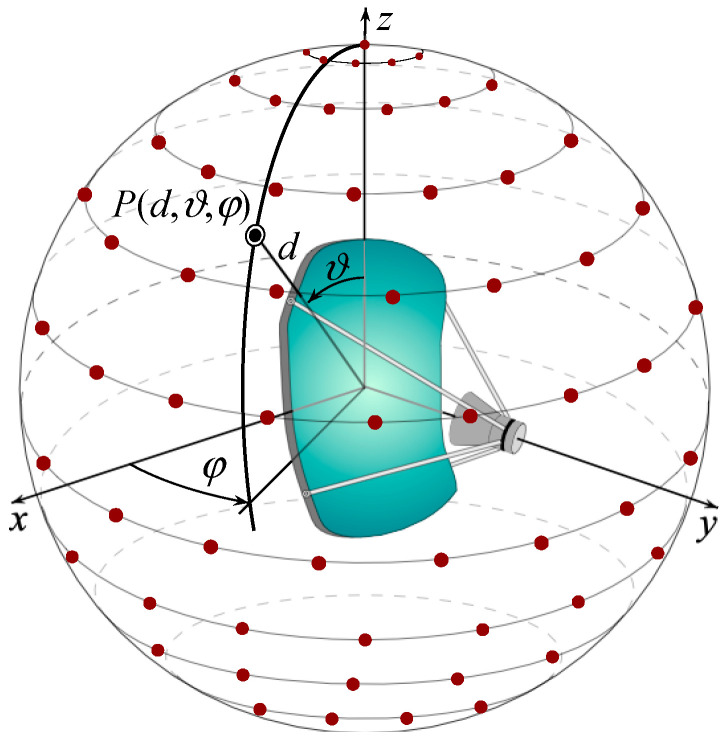
SNF scanning.

**Figure 2:**
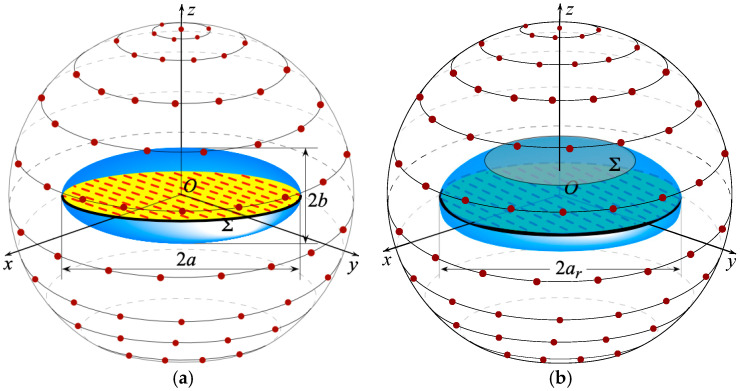
SNF scanning for quasi-planar AUTs. (**a**) oblate spheroidal modeling; (**b**) double bowl modeling.

The geometry of the problem shown in Figure 1 depicts an antenna to be characterized within an SNF facility. As can be seen, a spherical coordinate system (r,ϑ,φ), having its center coincident with the geometric center of the AUT, is introduced for individuating any given observation point *P* on a scan sphere of radius *d*, which surrounds the AUT. The scan sphere is supposed to be scanned by a small probe with very low directive characteristics. Note that such a type of probe ensures that the theoretical results in [37] can be also applied to the representation of the measured voltage, since its bandwidth is practically the same as the AUT field [47]. Thus, the development of the NR representation proceeds by first choosing the modeling surface Σ providing the best fitting of the AUT shape. To this end, either an oblate spheroid of semi-axes *a* and *b* (see Figure 2a) [11,17,27] or a convex surface called a double bowl (see Figure 2b) [11,25,28], obtained by joining two circular bowls with the same aperture radius $a_{r}$ and bending radii of the lower and upper arcs *c*′ and *c*, can be chosen for quasi-planar AUTs. Then, an optimal parameter *ξ* must be determined to represent any of the curves (parallels and meridians) describing the spherical surface, and an optimal phase factor ${\;e}^{j\gamma (\xi )}$ has to be singled out from the voltage expressions on them. This makes possible the introduction of the reduced voltage $\tilde{V}(\xi )=V(\xi ){\;e}^{j\gamma (\xi )}=V_{p,r}(\xi ){\;e}^{j\gamma (\xi )}$, where $V_{p}$ and $V_{r}$ refer to the voltage measured by the probe or the rotated probe. The so defined reduced voltage exhibits the property of being a spatially quasi band-limited function [11,37], so it can be successfully approximated with a band-limited function, provided that the bandwidth gets over a critical value $W_{\xi }$ [37]. Indeed, the introduction of an enlargement bandwidth factor χ′, a bit greater than one for electrically large AUTs, allows the effective control of the remaining approximation error.

The bandwidth, the phase factor, and the optimal parameter allowing the development of the NR voltage representation on a meridian can be obtained [11,37] by using the following relations:

(1) $$W_{\xi }=\frac{l\prime }{\lambda }$$

(2) $$\gamma =\left(\pi /\lambda \right)\left[R_{1}+s_{1}^{\prime }+R_{2}-s_{2}^{\prime }\right]$$

(3) $$\xi =\left(\pi /l\prime \right)\left[R_{1}+s_{1}^{\prime }-R_{2}+s_{2}^{\prime }\right]$$

In these relations, λ is the wavelength, l′ is the length of the curve *C*′, which is obtained by intersecting the meridian plane through *P* with Σ and depends on the choice of the antenna modeling [11,37]. As a consequence, when choosing the oblate spheroid, such a length is [11,17,27] $l\prime =4a\;E\left(\pi /2\left|\;\varepsilon^{2}\right.\right)$, wherein *E*(·|·) represents the second kind elliptic integral, ε=f/a being the spheroid eccentricity and 2*f* the focal distance. Whilst, when employing the double bowl modeling, it results [11,25,28] $l\prime =2\left[2a_{r}+\left(c+c\prime \right)\left(\pi /2-1\right)\right]$. Moreover, $R_{1,\;2}$ are the distances between *P* and the points $P_{1,\;2}$ of tangency on *C*′, $s_{1,\;2}^{\prime }$ are their curvilinear abscissas [11,37]. It must be stressed that, when dealing with the double bowl modeling, *γ* and *ξ* can be evaluated by substituting in (2) and (3) the proper values of $R_{1,\;2}$ and $s_{1,\;2}^{\prime }$, whose expressions change, according to geometric considerations, in dependence of where $P_{1,\;2}$ occur on moving *P*, and are explicitly reported in [11,25,28]. On the other hand, when adopting the oblate spheroidal modeling, it can be shown [11,37] that the curves at *ξ* = constant and *γ* = constant are, in any meridian plane, hyperbolas and ellipses confocal to the ellipse *C*′, and are functions only of the elliptic coordinates $u=(r_{1}-r_{2})/2f$, $v=(r_{1}+r_{2})/2a$, wherein $r_{1,2}$ are the distances from *P* to the foci. Their expressions, explicitly reported in [11,17,27], can be numerically evaluated through the *E*(·|·) function.

When considering a parallel at *ϑ*, it can be shown [11,37] that the phase function *γ* is constant and equal to that relevant to the meridian curve through any point *P* lying on it. Moreover, the azimuthal angle *φ* can be profitably employed to describe it, since, in such a case, any parameter proportional to the curvilinear abscissa *s* is optimal. As regards the corresponding bandwidth, the following results [11,37]:

(4) $$W_{\varphi }=\;\;\frac{\pi }{\lambda }\;\underset{z\prime }{\max}\left(R^{+}-R^{-}\right)=\frac{\pi }{\lambda }\underset{z\prime }{\max}\;\left(\sqrt{{\left(\rho +\rho \prime (z\prime )\right)}^{2}+{\left(z-z\prime \right)}^{2}}\right.\left.-\;\sqrt{{\left(\rho -\rho \prime (z\prime )\right)}^{2}+{\left(z-z\prime \right)}^{2}}\right)$$

wherein ρ′(z′) is the equation of Σ in cylindrical coordinates and ρ=dsinϑ, z=dcosϑ. It can be shown [11,37] that such a maximum is found on that portion of Σ which lies on the same side of the scan parallel with respect to the maximum transverse circumference of Σ. When choosing the oblate spheroidal modeling, as shown in [11,37], the bandwidth $W_{\varphi }$ particularizes as follows [17,27]:

(5) $$W_{\varphi }=\;\;\frac{2\pi \;a}{\lambda }\;\sin\vartheta_{\infty }$$

where $\vartheta_{\infty }=\sin^{-1}u$ is the zenithal angle of the asymptote to the hyperbola that passes through *P* [11,37]. The interested reader can find in [25,28] the analytical details concerning the evaluation of the maximum in (4) in the case of the double bowl modeling.

Regardless of the choice of the AUT modeling, the NF measurements are collected over a NR grid in which the sampling parallels are non-uniformly distributed in the *ϑ* parameter, but uniformly distributed in the *ξ* parameter with spacing Δξ given by the following:

(6) $$\Delta \xi =\frac{2\pi }{2N^{\prime \prime }\;+\;1}$$

wherein

(7) $$N\prime =\mathrm{Int}\left(\chi \prime W_{\xi }\right)+1;\;N^{\prime \prime }=\mathrm{Int}\left(\chi \;N\prime \right)+1$$

χ > 1 being an oversampling factor [11,37] and Int(*x*) denoting the integer part of *x*. The sampling points on each of them are equispaced by

(8) $$\Delta \varphi_{n}=\frac{2\pi }{2M_{n}^{\prime \prime }+1},\;n=1,\ldots ,N^{\prime \prime }$$

wherein

(9) $$M_{n}^{\prime \prime }=\mathrm{Int}\left(\chi \;M_{n}\right)+1;\;M_{n}^{\prime }=\mathrm{Int}\left[\chi^{*}W_{\varphi }(\xi_{n})\right]+1\;$$

and

$$\chi^{*}=\left\{\begin{array}{l} 1+(\chi \prime -1){\left[\sin\vartheta (\xi_{n})\right]}^{-2/3}\;\;\;\;\;\;\;\;\mathrm{double}\;\mathrm{bowl}\;\\ 1+(\chi \prime -1){\left[\sin\vartheta_{\infty }(\xi_{n})\right]}^{-2/3}\;\;\;\;\mathrm{oblate}\;\mathrm{spheroid} \end{array}\right.$$

is the azimuthal bandwidth enlargement factor [11,37].

Then, the probe voltage at a point P(ϑ,φ) on the scan sphere and, in particular, at those for the standard SNF raster grid [16] can be restored, from the NR samples collected at the points of the NR sampling grid, via the following OSI expansion [11,37]:

(10) $$V\left(\xi (\vartheta ),\;\varphi \right)=e^{-j\gamma (\xi )}\sum_{n=\;n_{0}-q+1}^{n_{0}+q}\left\{\;G\left(\xi ,\xi_{n},\bar{\xi },N,N^{\prime \prime }\right)\right.\sum_{m=m_{0}-p+1}^{m_{0}+p}\;\left.\tilde{V}\left(\xi_{n},\varphi_{m,n}\right)G\left(\varphi ,\varphi_{m,n},{\bar{\varphi }}_{n},M_{n},M_{n}\prime \prime \right)\right\}\;$$

As can be seen, relation (10) is a 2-D traveling expansion of the central type, that requires the 2q×2p NF samples closest to the output point *P*, specified by the indexes $n_{0}=\mathrm{Int}\left(\xi /\Delta \xi \right)$ and $m_{0}=\mathrm{Int}\left(\varphi /\Delta \varphi_{n}\right)$ along the meridians and the parallels, respectively. Moreover, $\tilde{V}\;\left(\xi_{n},\varphi_{m,n}\right)$ are the reduced voltages collected at the positions specified by [11,37]

(11) $$\xi_{n}=n\Delta \xi ;\;\varphi_{m,n}=m\Delta \varphi_{n}$$

and

(12) $$N=N^{\prime \prime }-N^{\prime };\;M_{n}=M_{n}^{\prime }-M_{n}^{\prime \prime }$$

(13) $$\bar{\xi }=q\Delta \xi ;\;{\bar{\varphi }}_{n}=p\;\Delta \varphi_{n}$$

The kernel of the OSI expansion (10), that allows one to minimise the truncation error for a reduced number of considered samples,

(14) $$G\left(\alpha ,\alpha_{k},\bar{\alpha },L,L^{\prime \prime }\right)=D_{L^{\prime \prime }\;}\left(\alpha \;-\;\alpha_{k}\right)\Omega_{\;L}\left[\left(\alpha \;-\;\alpha_{k}\right),\bar{\alpha }\right]$$

is given as the product [11,37] of the standard interpolation function for a closed domain, i.e., the Dirichlet function,

(15) $$D_{L^{\prime \prime }}\left(\alpha \right)=\frac{\sin\left[(2L^{\prime \prime }+1)\;\alpha /2\right]}{\left(2L^{\prime \prime }+1)\;\sin(\alpha /2\right)}$$

by the Tschebyscheff sampling window, capable of accelerating the convergence of the OSI expansion,

(16) $$\Omega_{\;L}\left(\alpha ,\bar{\alpha }\right)=\frac{T_{L}\left[2\cos^{2}\left(\alpha /2\right)/\cos^{2}\left(\bar{\alpha }/2\right)-1\right]}{T_{L}\left[\;2/\cos^{2}\left(\bar{\alpha }/2\right)-1\right]}$$

with $T_{L}[\cdot ]$ being the *L* degree Tschebyscheff polynomial.

## 3. Two-Step Procedure for Correcting 3-D Probe Mispositioning Errors

**Figure 3:**
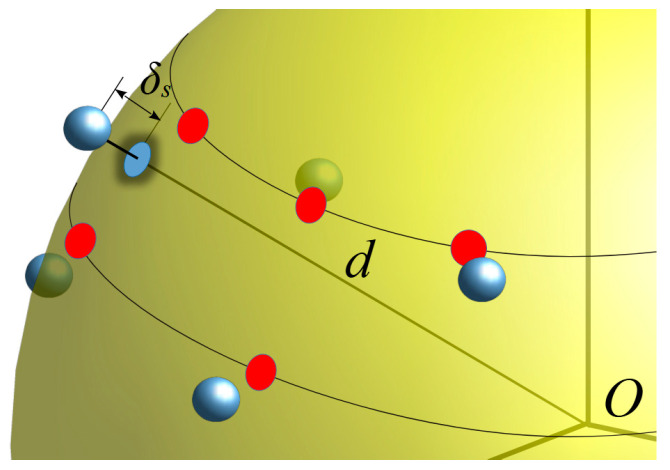
Relevant to the 3-D mispositioned samples.

**Figure 4:**
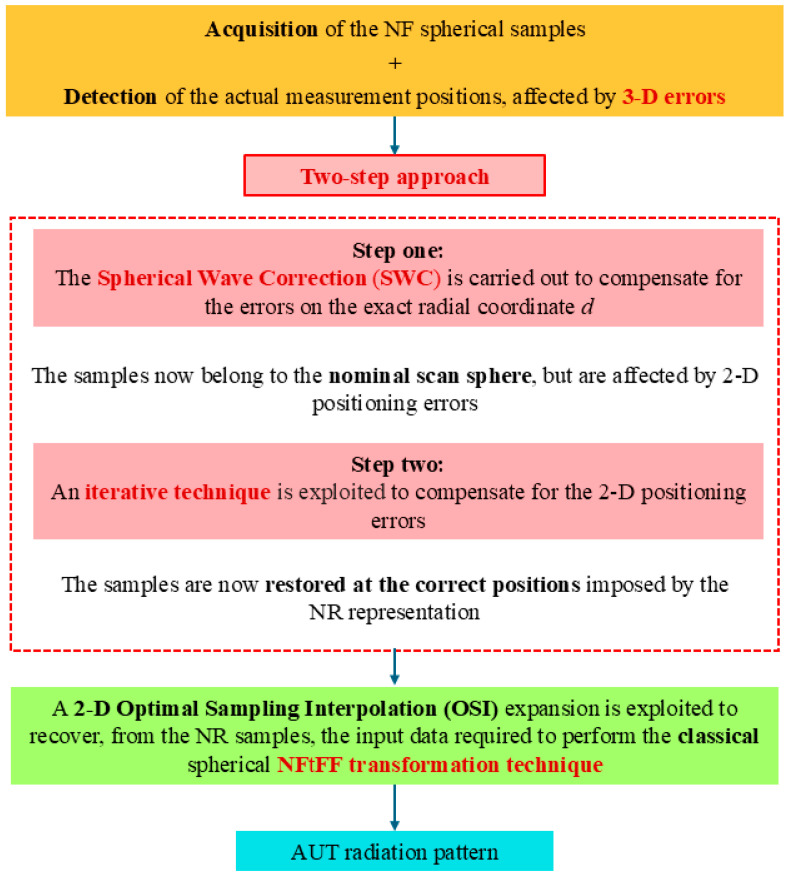
Flowchart of the two-step procedure.

In the following, it is assumed that, apart from the sample at the north pole, the prescribed points (10) of the NR grid cannot be accurately reached due to the limited accuracy of the positioners and controllers of the NF measurement system. Moreover, besides these 2-D positioning errors, all the acquired NF samples are also affected by a radial displacement (see Figure 3). According to such conditions, the NF measurements suffer from 3-D mispositioning errors, impairing the overall accuracy of the NF and FF recoveries. Fortunately, the exact positions of the acquiring probe may be detected by using laser devices and, hence, are assumed as known. This hypothesis allows one to formalize the problem at hand as two independent problems, one due to the deviations of the measurement points from the prescribed scan sphere and the other due to the 2-D positioning errors. These problems can be factorized and solved by devising a novel two-step correction procedure, which permits to restore of the NR samples at their exact positions, starting from those perturbed by the 3-D errors (see Figure 4). It proceeds by first compensating the phase errors introduced by the displacements of the sampling positions away from the nominal scan sphere. To this end, a phase correction, named SW correction to parallel the assumption behind the use of the wave expansion employed to evaluate the FF pattern, is here proposed to compensate the radial mispositioning of each NF sample. Let $\rho_{s}$, with $s=1,\ldots ,N_{T}$, be the shift of each sampling point from the scan sphere and $V(d+\rho_{s},\vartheta ,\varphi )$ denote each mispositioned voltage sample. To estimate the phase that the NF sample would have if it were correctly acquired on the set scan sphere, the SW phase correction step consists in multiplying $V(d+\rho_{s},\vartheta ,\varphi )$ by the phase factor $e^{j\;2\pi \;\rho_{s}/\lambda }$, namely

(17) $$V(d,\vartheta ,\varphi )=e^{j\;2\pi \;\rho_{s}/\lambda }V(d+\rho_{s},\vartheta ,\varphi )$$

At the end of this step, each voltage value recovered via (17) is now relative to a sampling point lying on the scan sphere, but it is still affected by a 2-D mispositioning error. Fortunately, two correction techniques able to face this kind of error in the NR framework are already available in the literature [45,46]. The former utilizes an iterative procedure, which converges only when there exists a biunique relationship associating each non-uniform NF sample, namely that at the incorrect sampling point to the corresponding one at the nearest point of the NR grid (uniform sample). The latter, based on singular value decomposition, can be adopted when the 2-D error compensation problem can be divided into two independent 1-D problems, thus reducing the computational burden. The distribution of the 2-D error affected samples, as a result of the first step, is better suited to be corrected by using the iterative based technique, since such an arrangement realistically matches the conditions for its application. Accordingly, the latter has been adopted to retrieve the NF samples at the correct sampling points of the NR grid.

To match the above assumption, let $\left(\eta_{\;k},\sigma_{j,k}\right)$ be the non-uniform sampling points and suppose they are in one-to-one correspondence with the nearest uniform ones $\left(\xi_{n},\varphi_{m,n}\right)$. The reduced voltage samples at $\left(\eta_{\;k},\sigma_{j,k}\right)$ can be related to those at $\left(\xi_{n},\varphi_{m,n}\right)$ through the OSI expansion (10), thus giving

(18) $$\tilde{V}(\eta_{k},\sigma_{j,k})=\sum_{n=\;n_{0}-q+1}^{n_{0}+q}\left\{G\left(\eta_{k},\xi_{n},\bar{\xi },N,N^{\prime \prime }\right)\right.\sum_{m=m_{0}-p+1}^{m_{0}+p}\;\left.\tilde{V}\;\left(\xi_{n},\varphi_{m,n}\right)G\left(\sigma_{j,k},\varphi_{m,n},{\bar{\varphi }}_{n},\;M_{n},M_{n}^{\prime \prime }\right)\right\}\;\;$$

This last relation can be conveniently written in matrix form as

(19) $$\underline{\underline{Z}}\;\underline{U}={\underline{U}}_{NU}\;\;$$

where $\underline{\underline{Z}}$ is an $N_{T}\times N_{T}$ sparse banded matrix, while $\underline{U}$ and ${\underline{U}}_{NU}$ denote the column vectors of the unknown uniform and known non-uniform samples, respectively.

The solution of relation (19) is then achieved by employing the iterative technique [45,46]. Specifically, it is first rearranged by subdividing $\underline{\underline{Z}}$ into its non-diagonal and diagonal parts, say $\underline{\underline{\Delta }}\;$ and ${\underline{\underline{Z}}}_{D}$. Then, by multiplying these terms by ${\underline{\underline{Z}}}_{D}^{\;-1}$, it follows that

(20) $${\underline{U}}^{\left(\nu \right)}\;={\underline{\underline{Z}}}_{D}^{\;-1}{\underline{U}}_{NU}-\;{\underline{\underline{Z}}}_{D}^{\;-1}\underline{\underline{\Delta }}\;{\underline{U}}^{\left(\nu -1\right)}={\underline{U}}^{\left(0\right)}-\;{\underline{\underline{Z}}}_{D}^{\;-1}\underline{\underline{\Delta }}\;{\underline{U}}^{\left(\nu -1\right)}$$

wherein ${\underline{U}}^{\left(\nu \right)}$ is the column vector of the NF samples at the correct points of the NR grid evaluated at the ν-th iteration.

The explicit formulation of (20) yields the following:

(21) $$\begin{array}{l} {\tilde{V}}^{\left(\nu \right)}\left(\xi_{n},\varphi_{m,n}\right)=\\ \;\frac{1}{DIV}\{\tilde{V}(\eta_{n},\sigma_{m,n})-\underset{\left(k\neq n)\;\;\wedge \;\;(j\neq m\right)}{\sum_{k=\;k_{0}-q+1}^{k_{0}+q}\sum_{j=\;j_{0}-p+1}^{j_{0}+p}}\;G\left(\eta_{n},\xi_{k},\bar{\xi },N,N^{\prime \prime }\right){\tilde{V}}^{\left(\nu -1\right)}\left(\xi_{k},\varphi_{j,k}\right)G(\sigma_{m,n},\varphi_{j,k},{\bar{\varphi }}_{j},M_{k},M_{k}^{\prime \prime })\} \end{array}$$

where

(22) $$\;\;DIV=\;\;G\left(\eta_{n},\xi_{n},\bar{\xi },N,N^{\prime \prime }\right)G\left(\sigma_{m,n},\varphi_{m,n},{\bar{\varphi }}_{n},\;M_{n},M_{n}^{\prime \prime }\right)$$

(23) $$j_{0}=\left\{\begin{array}{l} m\;\;\;\;\;\mathrm{if}\;\sigma_{m,n}\geq \;\varphi_{m,n}\\ m-1\;\;\;\;\;\mathrm{if}\;\sigma_{m,n}<\varphi_{m,n} \end{array}\right.;\;k_{0}=\left\{\begin{array}{c} n\;\;\;\mathrm{if}\;\;\;\eta_{n}\geq \;\;\xi_{n}\\ n-1\;\mathrm{if}\;\;\;\eta_{n}<\;\;\xi_{n} \end{array}\right.$$

It must be observed that the necessary conditions [45,46] for the convergence of the algorithm are that each main diagonal entry of the matrix $\underline{\underline{Z}}$ has non-zero magnitude and exceeds the magnitude of all other entries in the same row or column. The distribution of the 2-D error affected samples, as previously assumed, inherently satisfies these requirements, thus ensuring that the convergence criteria are fulfilled.

## 4. Test Results

Extensive numerical tests have been conducted to evaluate the feasibility of the devised two-step procedure to compensate for 3-D mispositioning errors, which compromise the characterization of a quasi-planar antenna through a SNF facility, which is assumed to be equipped with a *φ* positioner and an arc guiding the measuring probe. The reported results here are relevant to two different AUTs, each modeled by one of the two available modelings suitable for a quasi-planar antenna.

The first set of results concerns a uniform planar circular array (AUT_1) of radius 15λ whose elements are *y*-polarized elementary Huygens dipoles, placed on the plane *z* = 0 of the reference system and spaced by 0.41λ along *x* and 0.43λ along *y*. The NR sampling grid has been achieved by modeling such an AUT by a double bowl with $a_{r}$ = 15λ and *c*′ = *c* = 2.4λ. The radius *d* of the measurement sphere has been set equal to 25λ. 

The latter is relevant again to a uniform planar circular array of *y*-polarized elementary Huygens dipoles (AUT_2) with radius 14λ, whose spacings are now Δx=0.4λ and Δy=0.5λ. Such an AUT has been considered as enclosed in an oblate spheroid having the semi-axes *a* and *b* equal to 14λ and 2.5λ. The measurement sphere has its radius equal to 24λ.

The NF samples were simulated as measured by an X-band open-ended rectangular waveguide (WR-90) at the NR grid points, which, due to the limited resolution of the positioners (controllers) and mechanical inaccuracies of the arc, result to be corrupted by known 3-D position errors. To model these errors, the shift $\rho_{s}$, with $s=1,\ldots ,N_{T}$, of each sampling point from the scan sphere has been described by a uniform random variable in the range [–0.1λ, 0.1λ], whilst the shifts of their positions $\left(\eta_{\;k},\sigma_{j,k}\right)$ from the correct ones of the NR grid have been modeled as uniform random variables in the ranges [−0.333Δ*ξ*, 0.333Δ*ξ*] and $\left[-0.333\;\Delta \varphi_{n},0.333\;\Delta \varphi_{n}\right]$, respectively.

The feasibility of the proposed two-step procedure in compensating for even significant 3-D positioning errors can be verified by examining the resulting NF and FF reconstructions.

**Figure 5:**
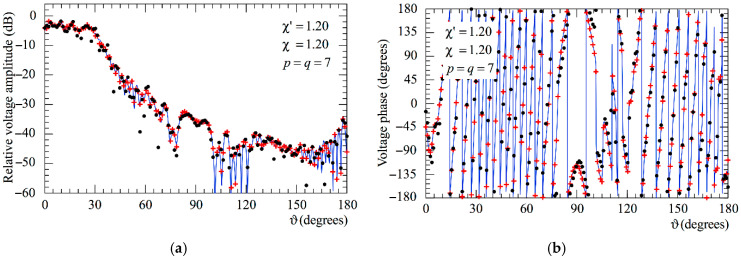
AUT_1. Voltage $V_{p}$ on the meridian at *φ* = 30°. ––––– exact. ++++ obtained from the mispositioning error affected NF samples via the two-step procedure. •   •   •   • attained from the positioning errors altered NF samples without performing the two-step procedure: (**a**) Amplitude; (**b**) Phase.

**Figure 6:**
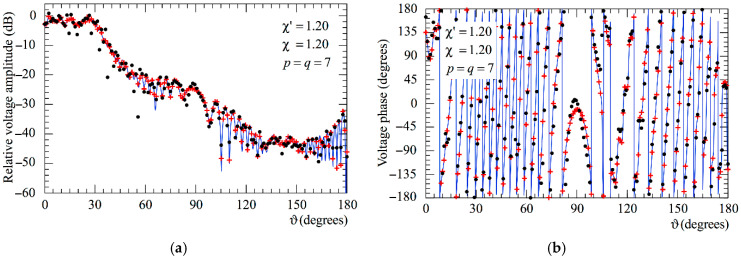
AUT_1. Voltage $V_{r}$ on the meridian at *φ* = 90°. ––––– exact. ++++ obtained from the mispositioning error affected NF samples via the two-step procedure. •   •   •   • attained from the positioning errors altered NF samples without performing the two-step procedure: (**a**) Amplitude; (**b**) Phase.

**Figure 7:**
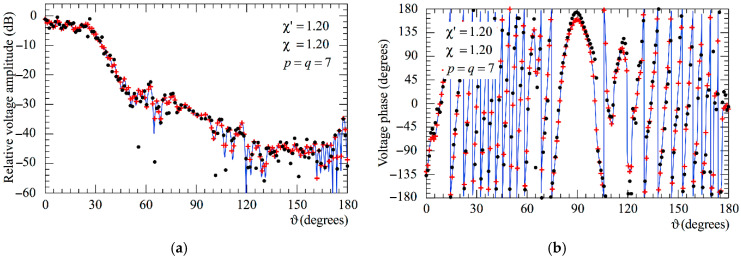
AUT_2. Voltage $V_{p}$ on the meridian at *φ* = 30°. ––––– exact. ++++ obtained from the mispositioning error affected NF samples via the two-step procedure. •   •   •   • attained from the positioning errors altered NF samples without performing the two-step procedure: (**a**) Amplitude; (**b**) Phase.

**Figure 8:**
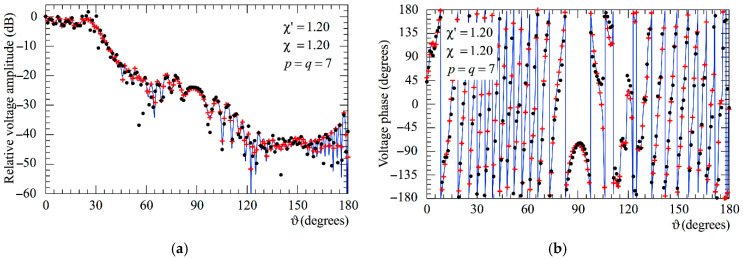
AUT_2. Voltage $V_{r}$ on the meridian at *φ* = 90°. ––––– exact. ++++ obtained from the mispositioning error affected NF samples via the two-step procedure. •   •   •   • attained from the positioning errors altered NF samples without performing the two-step procedure: (**a**) Amplitude; (**b**) Phase.

**Figure 9:**
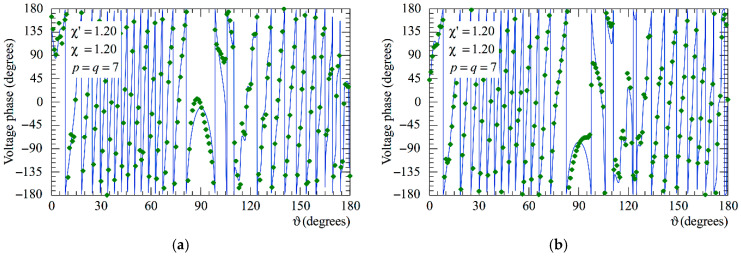
Phase of the voltage $V_{r}$ on the meridian at *φ* = 90°. ––––– exact. ◆◆◆◆ obtained from the mispositioning errors impaired NF samples by only applying the iterative scheme: (**a**) AUT_1; (**b**) AUT_2.

**Figure 10:**
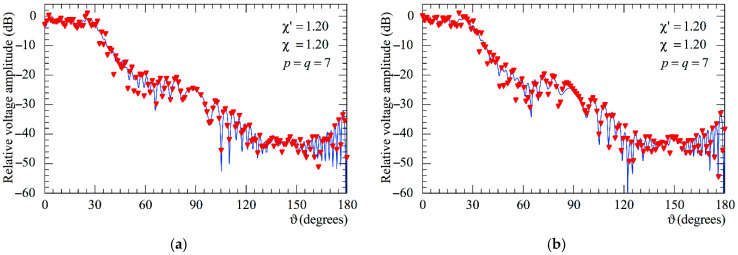
Amplitude of the voltage $V_{r}$ on the meridian at *φ* = 90°. ––––– exact. ▼▼▼▼ obtainedt from the mispositioning errors impaired NF samples by only applying the SW phase correction: (**a**) AUT_1; (**b**) AUT_2.

The following figures show the reconstructions of the amplitudes and phases of the voltages $V_{p}$ and $V_{r}$ along the meridians at *φ* = 30° and 90°, respectively, obtained by interpolating via (10) the NF samples corrected by using the two-step procedure as compared to the exact ones and to those attained by direct interpolation of the 3-D positioning errors corrupted NF data. In particular, Figure 5 and Figure 6 refer to the AUT_1 and Figure 7 and Figure 8 are relevant to the AUT_2. As can be seen, the two-step procedure, i.e., the SW phase correction in (17) plus the iterative technique in (21), is able to accurately correct the injected pessimistic 3-D positioning errors and, in both cases, allows one to efficiently reconstruct the NF behavior. It is noteworthy that 10 iterations are considered sufficient [45,46] for the convergence of the iterative algorithm (21). Some might wonder what happens if only one of the two steps of the developed procedure is employed. Well, Figure 9 and Figure 10 are relevant to these occurrences in the case of AUT_1 and AUT_2. In particular, the reconstructions of the voltage phase when adopting only the iterative algorithm (21) are reported in Figure 9a,b, whilst the recoveries of the voltage amplitude when using only the SW correction (17) are shown in Figure 10a,b. As can be seen by comparing these reconstructions with the exact patterns, it appears evident that the SW phase correction must necessarily be joint to the iterative technique to reach satisfactory results and to avoid a significant degradation of the accuracy. From a computational point of view, it is important to highlight that the use of the former step, i.e., the SW phase correction, is straightforward and requires very little time.

Furthermore, it has been shown [45,46] that the employment of the iterative procedure is also not time-consuming. In particular, the iteration procedure required only 8.37 s for the AUT_1 case and 7.02 s for the AUT_2 case on an old Macbook Air equipped with a 1.7 GHz Intel Core I7 processor.

To stress the robustness of the two-step procedure, the amplitudes and phases of the voltage samples, already impaired by 3-D positioning errors, have been altered also by a background noise, with arbitrary phase and amplitude limited to Δ*α*, and an uncertainty of Δ*A*_r_ in amplitude and Δ*η*_r_ in phase, thus simulating an actual measurement performed in an anechoic chamber. 

**Figure 11:**
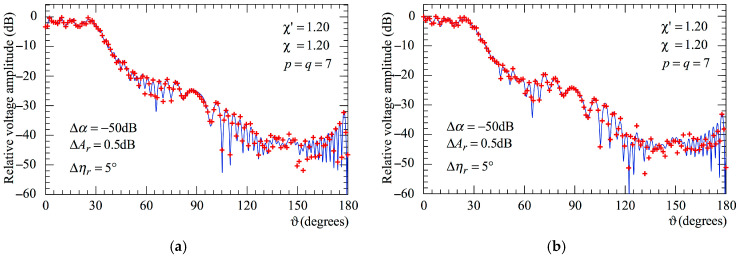
Amplitude of the voltage $V_{r}$ on the meridian at *φ* = 90°. ––––– exact. ++++ obtained from the mispositioning and measurement errors affected NF samples via the two-step procedure: (**a**) AUT_1; (**b**) AUT_2.

Figure 11a (AUT_1) and Figure 11b (AUT_2) show the reconstructions of voltage amplitude at *φ* = 90° obtained from these samples affected by both the error sources. As can be seen, the procedure results are stable even in a real measurement environment.

**Figure 12:**
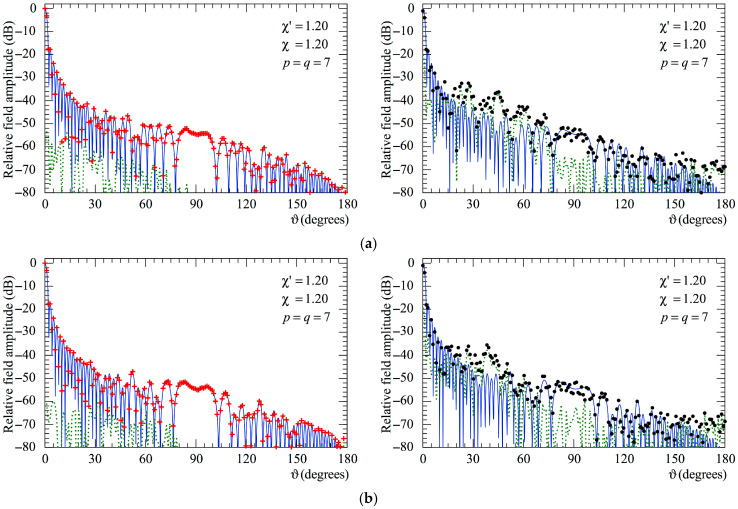
AUT_1. FF patterns in the principal planes. ––––– exact. ++++ obtained from the mispositioning errors corrupted NF samples via the two-step procedure. •   •   •   • achieved from the positioning errors altered NF samples without performing the two-step procedure. - - - - relative reconstruction error: (**a**) E-Plane; (**b**) H-plane.

**Figure 13:**
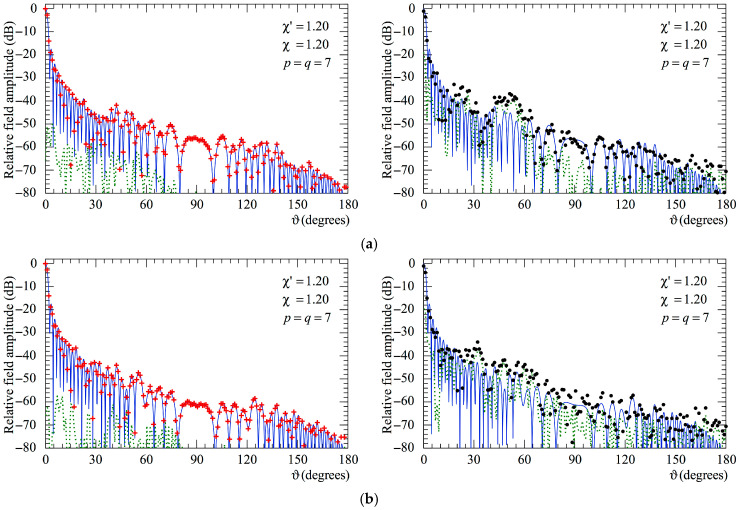
AUT_2. FF patterns in the principal planes. ––––– exact. ++++ obtained from the mispositioning errors altered NF samples via the two-step procedure. •   •   •   • achieved from the positioning error affected NF samples without performing the two-step procedure. - - - - relative reconstruction error: (**a**) E-Plane; (**b**) H-plane.

At last, the E- and H-plane FF patterns recovered from the 3-D positioning errors altered NF samples through the devised two-step procedure are compared in Figure 12 (AUT_1) and in Figure 13 (AUT_2) with the exact ones and with those obtained from the mispositioned samples without any correction. In these last figures, the corresponding reconstruction error is also shown (as a green dashed line). As can be seen, the results clearly show that the developed approach yields highly accurate FF reconstructions in both principal planes. Conversely, those achieved when it is not applied appear seriously degraded. 

**Figure 14:**
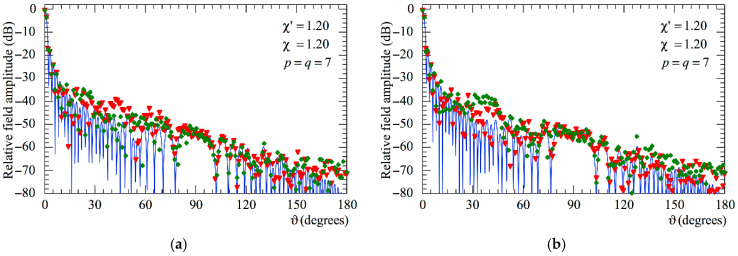
AUT_1. FF patterns in the principal planes. ––––– exact. ▼▼▼▼ obtained from the positioning errors altered NF samples by only applying the SW correction. ◆◆◆◆ obtained from the positioning error affected NF samples by only using the iterative technique: (**a**) E-Plane; (**b**) H-plane.

**Figure 15:**
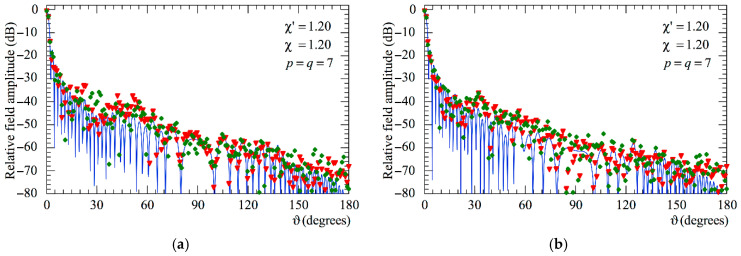
AUT_2. FF patterns in the principal planes. ––––– exact. ▼▼▼▼ obtained from the positioning error altered NF samples by only applying the SW correction. ◆◆◆◆ obtained from the positioning error affected NF samples by only using the iterative technique: (**a**) E-Plane; (**b**) H-plane.

Moreover, as can be seen from Figure 14 and Figure 15 relevant to the AUT_1 and AUT_2, respectively, also in this case, as previously observed for the NF results, it is necessary to employ both the SW phase correction and the iterative scheme in order to reach high accuracy and a reliable reconstruction of the AUT far field. 

Finally, it must be noted that the number of sampling points of the NR grid is 14,676 for the AUT_1 and 12,507 for AUT_2, hence, significantly smaller than those 23,544 and 20,200, respectively, which would be necessary when applying the classical spherical NFtFF transformation [16].

The interested reader can find further, although preliminary, numerical results on the application of the devised procedure for the compensation of 3-D probe positioning errors affecting the acquired samples in the NR spherical NFtFF transformations for quasi-planar AUTs in the conference papers [48,49].

## 5. Concluding Remarks

This paper concerns a feasibility study on the correction of 3-D probe positioning errors impairing the accuracy of the characterization of quasi-planar antennas from a NR number of voltage samples. To this end, a compensation procedure was properly developed. It proceeds through two stages: the first aims to compensate for the errors, due to the deviations of the NF samples from the set scan sphere, using a SW phase correction; then an iterative technique is employed to retrieve the correctly positioned NF data from those obtained at the first step, which results to be still impaired by 2-D mispositioning errors. The shown numerical results, relative to the characterization, from 3-D probe mispositioning errors affected NF data of two distinct AUTs, modeled using each of the two available modelings for quasi-planar antennas, fully assessed the feasibility of the devised procedure and the necessity to apply both the steps to obtain precise NF and FF recoveries. As a future work, experimental testing aiming to verify the practical feasibility of the technique will be carried out first at the antenna characterization laboratory of the collaborating partner companies and then at the NF measurement system of the University of Salerno, once the necessary upgrade needed to make it possible has been realized.

## Data Availability

Data are contained within the article.
